# Why Low Outflow Facility and Low Aqueous Production May Be Risk Factors in Endoscopic Cyclophotocoagulation: A Goldmann-Based Perspective

**DOI:** 10.7759/cureus.107217

**Published:** 2026-04-17

**Authors:** Masaki Tanito

**Affiliations:** 1 Department of Ophthalmology, Shimane University Faculty of Medicine, Izumo, JPN

**Keywords:** aqueous humor dynamics, endoscopic cyclophotocoagulation, goldmann equation, hypotony, intraocular pressure, outflow facility

## Abstract

Endoscopic cyclophotocoagulation (ECP) lowers intraocular pressure (IOP) by reducing aqueous humor production, yet the magnitude of its effect varies considerably among eyes and may occasionally lead to hypotony. Based on the Goldmann equation, in which IOP is expressed as IOP = episcleral venous pressure (EVP) + (aqueous humor production (F) − uveoscleral outflow (U)) / outflow facility (C), the pressure reduction induced by aqueous suppression can be approximated as ΔIOP (change in IOP) = dF (change in F) / C, when EVP and U are assumed to be constant. This relationship indicates that the IOP response to ECP depends not only on the degree of aqueous suppression but also strongly on the conventional outflow facility. A conceptual model assuming a fixed preoperative IOP demonstrates that eyes with low outflow facility exhibit a steeper pressure response to aqueous suppression and that eyes with low baseline aqueous production are mathematically linked to such low outflow states. These findings suggest that eyes characterized by both reduced aqueous production and impaired outflow may be particularly susceptible to excessive IOP reduction after ECP. Clinically, this includes conditions such as neovascular glaucoma with extensive angle closure, uveitic glaucoma, and glaucoma in elderly patients. The model presented is based on simplified assumptions, including constant episcleral venous pressure and uveoscleral outflow, and may not fully account for alterations in the pressure-flow relationship under pathological conditions; therefore, it should be interpreted with caution as a conceptual framework with limited physiological generalizability. However, the present conceptual framework provides a theoretical explanation for variability in ECP outcomes and suggests that securing a certain level of outflow facility prior to aqueous-suppressive procedures may be a reasonable strategy in selected cases.

## Editorial

Endoscopic cyclophotocoagulation (ECP) lowers intraocular pressure (IOP) by selectively photocoagulating the ciliary processes and thereby reducing aqueous humor production. Because this procedure acts primarily on aqueous inflow rather than enhancing conventional outflow pathways, its mechanism differs fundamentally from trabecular procedures, filtration surgery, or subconjunctival drainage devices. ECP has therefore been widely adopted as an adjunct to cataract surgery or as a treatment option across various stages of glaucoma. Clinical studies have demonstrated its usefulness both as a minimally invasive glaucoma surgery (MIGS)-type procedure and as a treatment for refractory glaucoma [1-4]. Nevertheless, the magnitude of IOP reduction after ECP varies considerably among eyes. In some patients, the pressure decrease is modest, whereas in others the reduction is substantial and occasionally accompanied by postoperative hypotony. In this editorial, based on the principle that ECP lowers IOP by reducing aqueous inflow [5], the factors that determine individual responsiveness are considered.

One possible explanation lies in the relationship between aqueous humor production and aqueous outflow described by the Goldmann equation [6]. Aqueous humor dynamics are commonly expressed as follows: F = C(IOP - EVP) + U, where F represents aqueous humor production, C is the conventional outflow facility, IOP denotes intraocular pressure, EVP represents episcleral venous pressure, and U represents uveoscleral outflow. Rearranging this equation yields IOP = EVP + (F − U) / C, indicating that IOP is determined by the balance between aqueous inflow and total outflow scaled by the conventional outflow facility. Because ECP primarily reduces aqueous humor production, the postoperative pressure after ECP can be expressed by replacing F with (F - dF), where dF denotes the reduction in aqueous production induced by the procedure. Substituting this term gives IOP_post = EVP + ((F_pre − dF) − U) / C, which leads to the pressure change: ΔIOP = IOP_pre − IOP_post = dF / C when episcleral venous pressure and uveoscleral outflow are assumed to remain constant (IOP_pre and IOP_post represent the IOP before and after ECP, respectively). This expression indicates that the pressure reduction per unit aqueous suppression is inversely proportional to the conventional outflow facility.

To illustrate this relationship conceptually, a simple mathematical simulation can be constructed. In the present conceptual model, preoperative IOP was assumed to be 40 mmHg in order to represent markedly elevated pressure frequently encountered in uncontrolled glaucoma. Episcleral venous pressure was assumed to be 8 mmHg, and uveoscleral outflow was assumed to be 0.5 μL/min, which is within the commonly reported physiological range [7,8]. Using the Goldmann equation, baseline aqueous humor production can, therefore, be expressed as F = C(IOP - EVP) + U.

**Figure 1:**
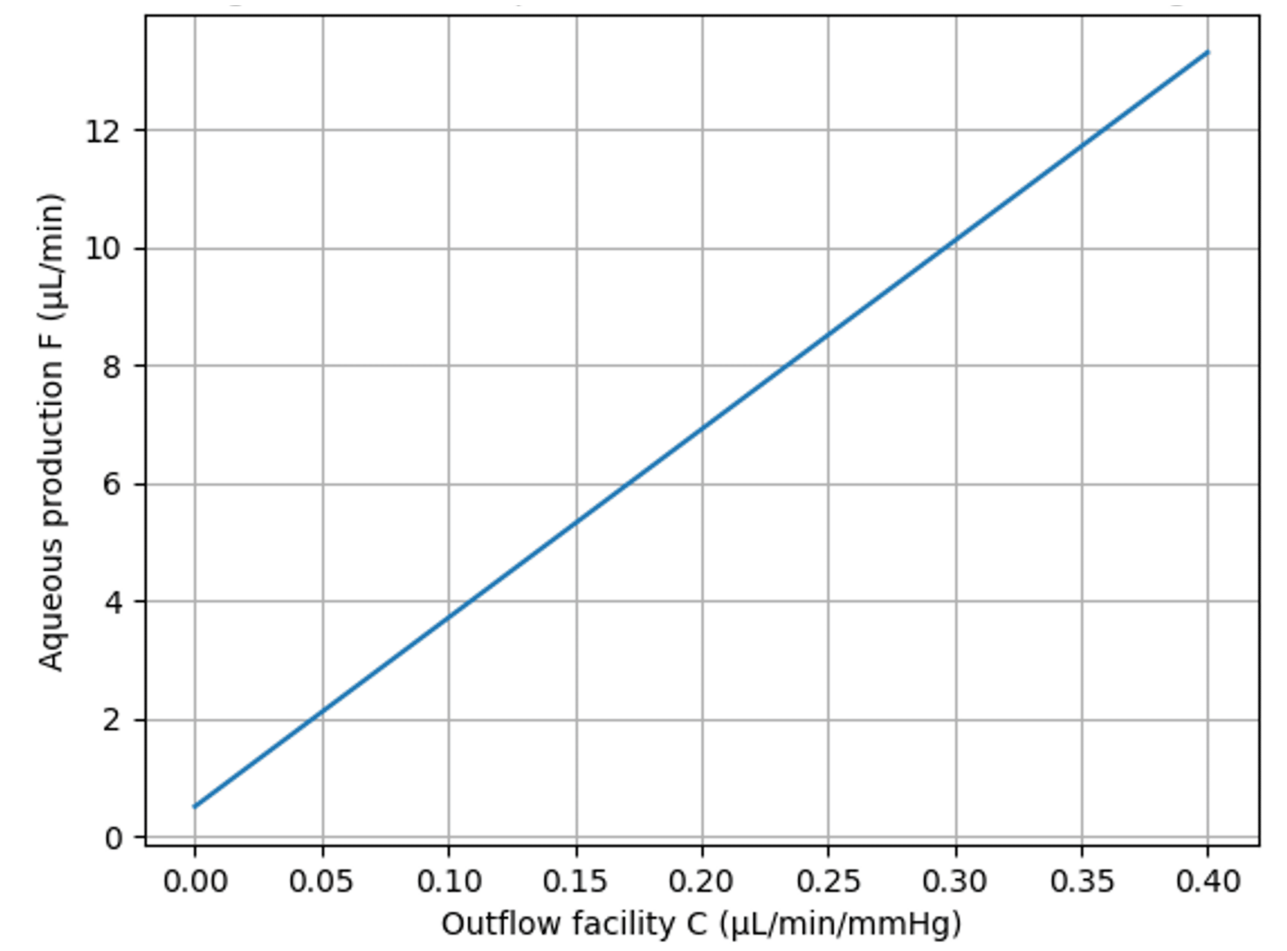
Relationship between aqueous humor production and conventional outflow facility at a fixed IOP. The relationship between aqueous humor production (F) and conventional outflow facility (C) was derived from the Goldmann equation [6], including uveoscleral outflow (F = C(IOP − EVP) + U). In this conceptual model, IOP was assumed to be 40 mmHg, episcleral venous pressure (EVP) was assumed to be 8 mmHg, and uveoscleral outflow (U) was assumed to be 0.5 μL/min. Under these conditions, aqueous humor production (F) increases linearly with increasing outflow facility (C) according to the equation F = 32C + 0.5. This figure was generated using ChatGPT-5.3 (OpenAI, San Francisco, California) and has not been previously published.

Under the assumption of a fixed IOP of 40 mmHg, this relationship simplifies to F = 32C + 0.5, indicating that aqueous humor production increases linearly with increasing outflow facility. This relationship is illustrated in Figure 1, which shows the theoretical association between aqueous humor production and conventional outflow facility when IOP is fixed at 40 mmHg. As the outflow facility increases, greater aqueous production is required to maintain the same IOP. Conversely, eyes with extremely low outflow facility require only small amounts of aqueous inflow to sustain high pressure. This indicates that even when the intraocular pressure is the same at 40 mmHg, there exist cases with completely different balances between F and C, such as eyes with preserved outflow facility in open-angle glaucoma and eyes with nearly absent outflow facility in circumferential angle closure.

**Figure 2:**
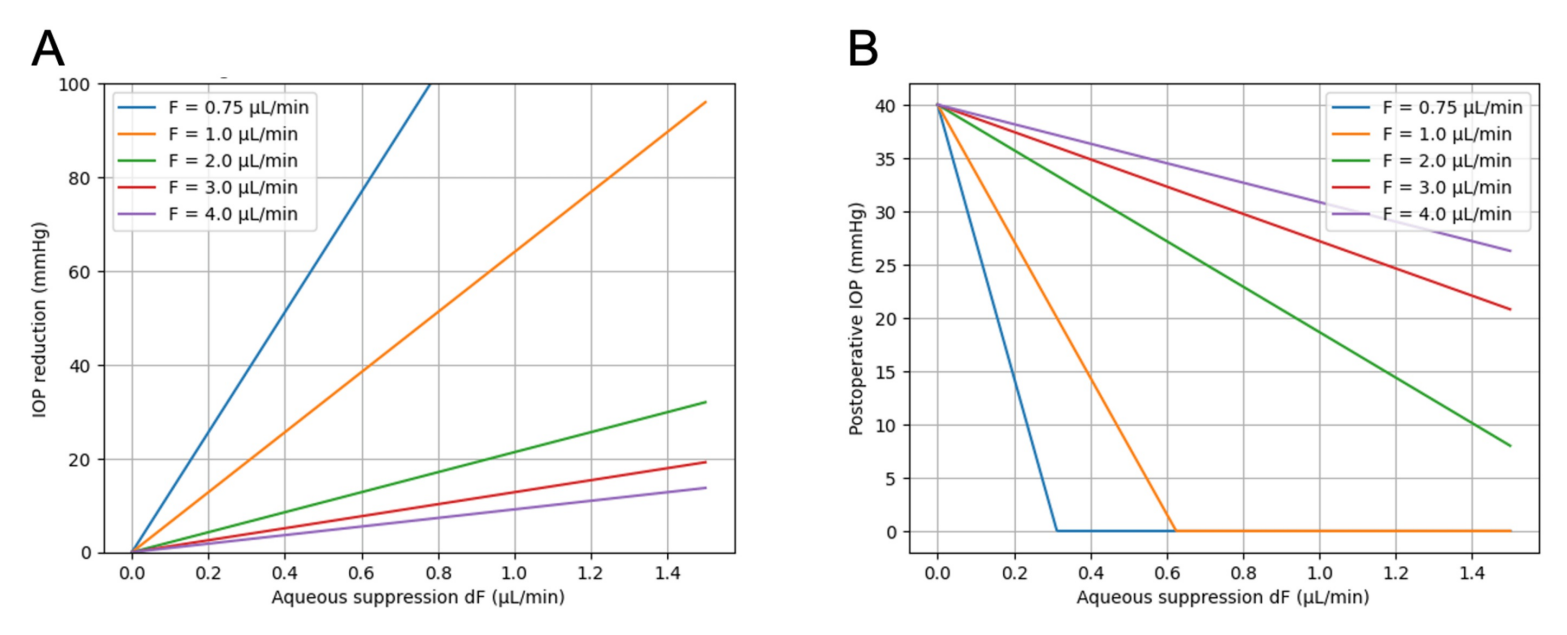
Effect of aqueous suppression on IOP reduction in eyes with different baseline aqueous humor production. (A) Modeled IOP reduction as a function of aqueous humor suppression (dF) for different baseline aqueous production levels (F = 0.75–4.0 μL/min). (B) Modeled postoperative IOP as a function of aqueous suppression under the same conditions. This figure was generated using ChatGPT-5.3 (OpenAI, San Francisco, California) and has not been previously published.

The effect of aqueous suppression on IOP can then be examined under different physiological assumptions. Figures 2A, 2B show the modeled relationship between aqueous suppression and pressure reduction in eyes with different baseline aqueous humor production levels. For each value of F, the corresponding outflow facility was calculated using the Goldmann equation: C = (F - U) / (IOP - EVP) [6]. Because the pressure response to aqueous suppression is given by ΔIOP = dF / C, eyes with smaller baseline aqueous production (and therefore smaller outflow facility) demonstrate a steeper pressure response. As a result, a relatively small reduction in aqueous humor production can produce a large decrease in intraocular pressure in such eyes. In contrast, eyes with larger baseline aqueous production require greater suppression of aqueous inflow to achieve the same pressure reduction.

**Figure 3:**
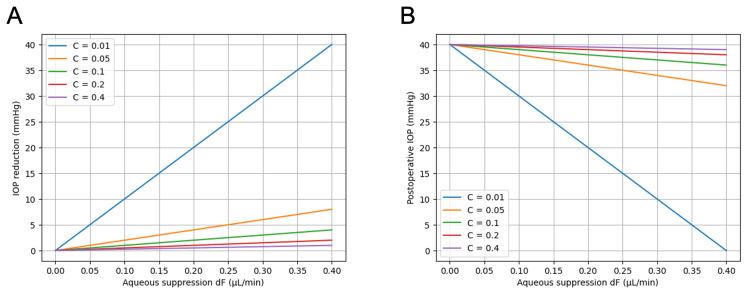
Effect of aqueous suppression on IOP reduction in eyes with different conventional outflow facility. (A) Modeled IOP reduction as a function of aqueous suppression for different outflow facility values (C = 0.01–0.40 μL/min/mmHg). (B) Modeled postoperative IOP as a function of aqueous suppression under the same conditions. This figure was generated using ChatGPT-5.3 (OpenAI, San Francisco, California) and has not been previously published.

The same phenomenon can also be viewed from the perspective of the outflow facility. Figures 3A, 3B illustrate the relationship between aqueous suppression and pressure reduction in eyes with different values of conventional outflow facility. When the outflow facility is low, the slope of the pressure response curve becomes steep, meaning that small changes in aqueous production can produce large changes in IOP. In eyes with relatively preserved outflow facility, the pressure response curve becomes shallower, and the same degree of aqueous suppression results in a smaller pressure reduction.

Taken together, these conceptual simulations highlight an important implication of the Goldmann equation: the pressure response to aqueous suppression depends strongly on the underlying outflow facility. Because the relationship ΔIOP = dF / C indicates that pressure reduction per unit aqueous suppression increases as outflow facility decreases, eyes with severely impaired outflow may exhibit a larger pressure response to the same degree of aqueous suppression. In such eyes, the effective therapeutic window for aqueous-suppressive procedures such as ECP may be narrower. A modest reduction in aqueous humor production could theoretically produce a large decrease in IOP, whereas in eyes with relatively preserved outflow facility, the same treatment may result in only moderate pressure reduction. Clinically, the most undesirable complication of ECP is persistent hypotony. The present modeling suggests that, particularly in eyes in which aqueous production is already reduced but IOP remains elevated due to markedly impaired outflow facility, ECP may lead to excessive IOP reduction. Such cases include neovascular glaucoma with circumferential angle closure, uveitic glaucoma, and glaucoma in elderly patients. This model therefore indicates that, in such eyes, it may be preferable to first secure a certain level of outflow facility with procedures such as tube shunt surgery, and to consider ECP only if elevated IOP persists thereafter.

It should be emphasized that the present analysis represents a conceptual mathematical illustration rather than a physiological simulation. Aqueous humor production itself shows partial pressure dependence, which may limit further pressure reduction at low IOP levels. Uveoscleral outflow may vary among individuals and may also change after ocular surgery or inflammation. In addition, biological responses of the ciliary body to photocoagulation may differ between eyes. These factors may modify the pressure response predicted by the simplified Goldmann relationship [9,10]. In addition, neovascular glaucoma and uveitic glaucoma are conditions in which, beyond differences in the balance between aqueous humor production and outflow, pathological changes in the composition of the aqueous humor, such as inflammation, elevated protein levels, and the presence of blood components, may also alter the pressure-flow relationship, making the response to ECP even more difficult to predict. Nevertheless, the conceptual framework presented here may help explain the variability in clinical outcomes observed after cyclodestructive procedures and provides a simple theoretical basis for understanding how aqueous suppression interacts with aqueous outflow.

In summary, a conceptual interpretation based on the Goldmann equation suggests that the IOP response to ECP depends strongly on the conventional outflow facility. Eyes with markedly reduced outflow facility may experience a larger pressure decrease for the same degree of aqueous suppression. Recognition of this relationship may help clinicians better understand variability in postoperative pressure responses following ECP and highlights the importance of considering aqueous outflow characteristics when interpreting the effects of aqueous-suppressive interventions.
